# Exoscope for revision of right lateral femoral cutaneous nerve decompression

**DOI:** 10.3171/2023.10.FOCVID23162

**Published:** 2024-01-01

**Authors:** Matthew C. Findlay, Spencer Twitchell, Mark A. Mahan

**Affiliations:** Department of Neurosurgery, Clinical Neurosciences Center, University of Utah, Salt Lake City, Utah

**Keywords:** lateral femoral cutaneous nerve, decompression, exoscope, meralgia paresthetica

## Abstract

The exoscope serves as a valuable addition or alternative to traditional microscope systems in surgery, offering 3D visualization and magnification with enhanced maneuverability. In lateral femoral cutaneous nerve decompression for meralgia paresthetica, the exoscope is effective in identifying strictures of neural compression and minimizing iatrogenic nerve damage that may lead to improved pain management outcomes for patients. In this report, the specific case presented showcases how the exoscope aided in surgical decompression of the lateral femoral cutaneous nerve of a patient with refractory meralgia paresthetica with remote previous decompression and resultant scarring.

The video can be found here: https://stream.cadmore.media/r10.3171/2023.10.FOCVID23162

## Transcript

This is Mark Mahan from the University of Utah, presenting a case of a revision lateral femoral cutaneous nerve decompression using the exoscope.

### 0:29 Patient History.

Our patient is a 69-year-old woman with a remote history of prior right- and left-sided lateral femoral cutaneous nerve decompressions as well as prior lumbar spine surgery. She presented to our clinic with progressively worsening bilateral lateral thigh pain and numbness consistent with recurrence of her meralgia paresthetica and was particularly severe on her left side.^1^ She had undergone a diagnostic block using ultrasound guidance, which had provided substantial relief of her symptoms.

On review of the patient’s MRI of the lumbar spine, there was no evident pathology involving either the L2 or L3 nerve root. These are the nerve roots that need to be evaluated carefully to ensure that the prior lateral femoral cutaneous nerve decompression was not a failed diagnosis, and, in fact, that the pathology arises from the spine. In this case, it was clear that there was no spinal pathology, and this is most likely due to either incomplete or recurrent lateral femoral cutaneous nerve entrapment.

And based on both the consistency of her symptoms with regard to mapping out the territory of the lateral femoral cutaneous nerve in her clinical diagram, as well as her response to localized injection under ultrasound guidance, we recommended consideration of repeat decompression of the lateral femoral cutaneous nerve.^2–6^

### 1:44 Rationale for Exoscope.

At the time, we decided to proceed with an exoscope approach for revision decompression. Our thinking was that a revision surgery requires microscopic visualization in order to be able to differentiate the difference between pale scar tissue and pale nervous tissue,^7–9^ and the illumination and magnification is absolutely necessary to be safe in distinguishing the two tissue types. Also, I tend to prefer a smaller skin incision for patients who have morbid obesity, particularly because of the fold of the inguinal crease creates an environment that is moist and difficult to heal, and a transverse skin incision heals better across a mobile segment of the hip. So, when using a small skin incision, we have to retract it both proximal distally throughout the case to visualize the area of pathology, and that assistant needs to know where to visualize the tissue. The exoscope also provides delightful surgeon ergonomics. The camera is placed approximately at the level of the surgeon’s shoulder, and the video monitor is placed directly across the patient, allowing the surgeon to stare directly ahead at the operative field.

The exoscope provides an ability for the assistant to know the pathology being treating at that time during the surgery, because they’re seeing exactly what the surgeon is seeing at the time of surgery.

### 3:03 Patient Positioning.

Once the patient was intubated in a supine position, we palpate the anterior superior iliac spine, mark it on the skin, and then use an ultrasound to map out the course of the lateral femoral cutaneous nerve, drawing it out with a surgical ink so that we can facilitate our approach.

### 3:19 Opening and Approach.

As you’ll see on the surgical approach here, the prior skin incision is marked with dotted ink, and our approach is just beneath it but above the inguinal crease. The ink mark for the nerve is also visualized on the left side of the screen, demonstrating where we have previously visualized the lateral femoral cutaneous nerve on ultrasound imaging. The next step in the procedure after the skin incision is to simply sweep the subcutaneous fat to visualize the deep fascia underneath and then divide the deep fascia using bipolar scissors.

I prefer to utilize bipolar scissors, which coagulate while cutting, which leads to less bleeding during surgery. The tips of the scissors are not as fine as fine tenotomies, but this can also be to advantage because they are about the same diameter as a hemostat for dissection.

We then rapidly progress down to the investing fashion of the tensor fascia lata and the sartorius and then find the fat pocket between the two muscles. Once that fat pocket is identified, open up this tissue, which in this case is a bit scarred, and be able to identify the nerve on the lateral margin of this fat pocket. After externally neuralizing the nerve, we’re able to place a vessel loop around the nerve to be able to manipulate it and maintain control.

### 4:34 Decompression.

We’ll then divide the superior fascia of the inguinal crease and then trace the nerve proximally into the inguinal canal. Again, this is a scarred area, and there’s slightly more scar tissue along the course of the lateral femoral cutaneous nerve. We will then identify and divide the deep layer of the inguinal fold with bipolar scissors and direct visualization above the nerve.

The exoscope provides 3D visualization at this point, which we can gauge depth as we enter into the pelvis. Then we’ll just continue to free up the nerve, and you can see there’s increased scar tissue on the lateral margin here, which is then divided again with bipolar scissors, and then on the medial margin, freeing up the scar tissue. Please note that the scar tissue around the lateral femoral cutaneous nerve is essentially precisely at the inguinal fold, meaning that the prior surgery did not address the majority of the pathology, which is at the inguinal fold and where the nerve starts to dive inside the pelvis. Again, the exoscope provides us an ability to peer into the pelvis. Once the nerve is circumferentially freed, you can see the pseudoneuroma and swelling of the nerve, and we’ll then identify the ligamentous ridge underneath the nerve and bipolar coagulate it and start to divide that ligamentous ridge to provide 360° decompression of the fascia around the nerve.

Here you can then identify the most deep portion and most proximal portion of the inguinal ligament by the assistant pulling on and retracting the tissues cranially. We’ll then be able to look inside the pelvis and make sure that the nerve is completely free on all sides, including the deep and back side of the nerve. At this point, we can visualize fibers of the iliacus muscle, which allows us to know the decompression is complete. In the final perspective of the nerve, you can see that it’s surrounded by muscle tissues and not fascial ridges.

### 6:23 Closure.

The operative field is lavaged aggressively with normal saline to make sure that there’s not any substantial bleeding after the surgery and that the skin is closed only at the superficial layer, with application of Steri-Strips and Dermabond to limit skin tension and facilitate appropriate wound closure.

## Acknowledgments

We thank Vance Mortimer for preparation of the video and Kristin Kraus for editorial assistance.

## Disclosures

Dr. Mahan reported personal fees from joimax and Renerva, and grants from Boston Scientific, Orthofix, and Nevro, outside the submitted work.

## Author Contributions

Primary surgeon: Mahan. Editing and drafting the video and abstract: all authors. Critically revising the work: all authors. Reviewed submitted version of the work: all authors. Approved the final version of the work on behalf of all authors: Mahan. Supervision: Mahan.

## Supplemental Information

### Patient Informed Consent

The necessary patient informed consent was obtained in this study.
